# Cytotoxicity of Two Resin-Based Sealers and a Fluoride Varnish on Human Gingival Fibroblasts

**Published:** 2015-03-18

**Authors:** Masoud Parirokh, Farshid Reza Forghani, Hamzeh Paseban, Saeed Asgary, Sara Askarifard, Saeed Esmaeeli Mahani

**Affiliations:** a *Kerman Neuroscience Research Center, Institute of Neuropharmacology, Kerman University of Medical Sciences, Kerman, Iran; *; b * Oral and Dental Diseases Research Center, Kerman University of Medical Sciences, Kerman, Iran; *; c * Iranian Center for Endodontic Research, Research Institute of Dental Sciences, Dental School, Shahid Beheshti University of Medical Sciences, Tehran, Iran*

**Keywords:** Cytotoxicity, Duraflur, Human Gingival Fibroblast, Methyl-thiazol-tetrazolium Assay, MTT Assay, Root Canal Sealer, Varnish

## Abstract

I**ntroduction: **Assessment of cellular cytotoxicity is a regular method for evaluating the biocompatibility of novel materials. In a recent study, 5% fluoride varnish (Duraflur) has shown reasonable sealing ability and coverage of root canal walls when used as a sealer. The aim of the present study was to compare the cytotoxicity of Duraflur varnish with two popular commonly used root canal sealers (AH-Plus and AH-26) on human gingival fibroblasts (HGF). **Methods and Materials: **The HGFs were incubated with different concentrations (1/2, 1/4, and 1/8) of AH-plus, AH-26, and Duraflur varnish for 24 h. The percentage of cell viability was assessed with methyl-thiazol-tetrazolium (MTT) assay. The data was analyzed using the one-way ANOVA followed by Student-Newman-Keuls test. The level of significance was set at 0.001. **Results: **MTT assay showed that higher concentrations of the tested materials resulted in lower viability of HGFs. AH-Plus showed significantly greater cell viability compared to AH-26 at all dilutions (*P*<0.001); however, no significant difference was found between Duraflur and AH-Plus in terms of cell viability at 1/8 dilution (*P*>0.001). Duraflur showed significantly higher cell viability compared to AH-26 except at 1/2 dilution (*P*<0.001). **Conclusion: **Although Duraflur varnish had better biocompatibility compared to AH-26, it should still be evaluated with further biocompatibility tests such as intraosseous and subcutaneous implantation.

## Introduction

One of the most important steps in endodontic practice is to seal the root canal space following cleaning and shaping [1]. Gutta-percha is the material of choice for this purpose [2]. However, as it is a solid material it must be used with an appropriate root canal sealer to improve obturation quality. An ideal root canal sealer should be nontoxic, dimensionally stable, biocompatible, radiopaque, and have a known solvent [3, 4]. So far, no root canal sealer has been introduced with all above mentioned properties. Therefore, introducing a new root canal sealer with reasonable sealing ability and biocompatibility is still the subject of ongoing studies [5].

Apart from sealing ability, root canal filling materials should have biocompatibility because they either intentionally or advertently may penetrate periradicular tissues and result in adverse inflammatory reactions [6]. A recent microleakage and scanning electron microscopy (SEM) study by similar authors, has shown that tooth varnish containing 5% fluoride (Duraflur) has reasonable sealing ability compared to AH-26 root canal sealer [7].

Several biocompatibility tests have been introduced for evaluating novel root canal filling materials such as cell toxicity, intraosseous and subcutaneous implantations [4, 8]. The aim of the present *in vitro *study was to compare the cellular toxicity of Duraflur varnish with two commonly used root canal sealers namely AH-26 and AH-Plus.

**Figure.1:**
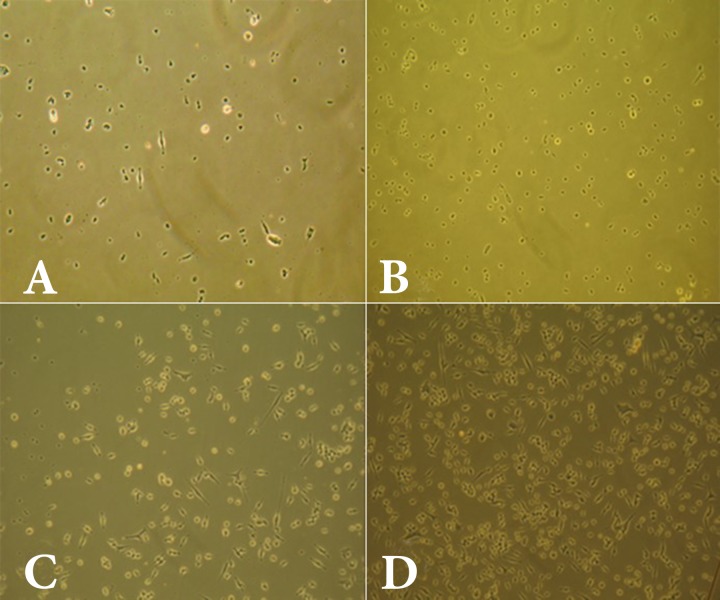
Cell viability among various dilutions of the AH-26 root canal sealer; *A)* 1/2 dilution; *B)* 1/4 dilution; *C)* 1/8 dilution; *D)* control

## Materials and Methods

For evaluating the cytotoxicity, a cell culture medium consisting of penicillin-streptomycin solution, trypsin, EDTA, fetal bovine serum (FBS) (PAA, Pasching, Austria) and heat-inactivated horse serum (HS) (Biosera Co., East Sussex, UK) was used. Normal human gingival fibroblasts (HGF) (line-PI1) were obtained from National Cell Bank of Iran (NCBI)(Pasteur Institute, Tehran, Iran). Cells were cultured in Dulbecco’s modified Eagle’s medium (DMEM; Gibco Laboratories, Grand Is., NY, USA) supplemented with 10% FBS, penicillin (100 U/ml) and streptomycin (100 µg/ml). They were then kept at 37^°^C in an atmosphere containing 5% CO_2_. After two passages, the cells were plated at the density of 5000 per well in a 96-well microplate for the methyl-thiazol-tetrazolium (MTT) assay. 

Then the cells were incubated with AH-Plus (Dentsply, Tulsa Dental, Tulsa, OK, USA), AH-26 (Dentsply, Tulsa Dental, Tulsa, OK, USA) and Duraflur (Pharmascience, Montreal, Québec, Canada) that were prepared as follows: freshly mixed materials were packed in glass rings (4 mm in height and 10 mm in diameter) and were left to set for 24 h at 37^º^C in a humidified chamber. Each sample was eluted in 10 mL of culture medium for 1 day in 5% CO_2_ at 37^º^C. The medium was then collected into sterile syringes at the end of this procedure and passed through a 0.22-μm filter. 

Finally various concentrations (1/2, 1/4 and 1/8) of this extraction media were prepared as follows: 100 μL medium (without test material) and 100 µL medium containing test materials, were added to obtain final volume of 200 µL to prepare 1/2 concentrations of the materials. For 1/4 and 1/8 dilutions, the same process was repeated.

**Figure 2:**
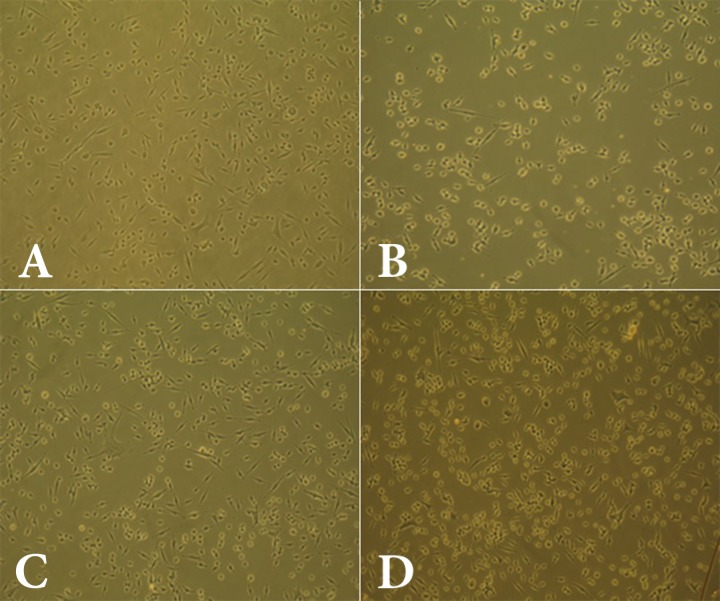
Cell viability among various dilutions of the AH-Plus root canal sealer; *A)* 1/2 dilution; *B)* 1/4 dilution; *C)* 1/8dilution; *D)* control

Cell viability assay and optical density (OD) of the groups were evaluated as follows: cellular viability was assessed by the reduction of yellow tetrazolium MTT [2-(4,5-dimethylthiazol-2-yl)-2,5-diphenyltetrazolium bromide] to formosan which is purple in color. The MTT solution was reduced by metabolically active cells, in part by the action of dehydrogenase enzymes, to generate reducing equivalents such as NADH and NADPH. The resulting intracellular formosan could be solubilized and quantified by spectrophotometric means. MTT was dissolved in phosphate-buffered saline (PBS) and added to the culture at final concentration of 0.5 mg/mL. After incubation for 2 h at 37^°^C, the media were carefully removed and 100 µL DMSO was added to each well, and the OD values were determined by spectrophotometry at 490 nm with microplate reader (ELX808 absorbance microplate reader; BioTek Instruments Inc., Winooski, VT, USA). Results were expressed as percentages of control.

The data were analyzed using the one-way ANOVA followed by the Student-Newman-Keuls test. The level of significance was set at 0.001.

## Results

**Figure 3:**
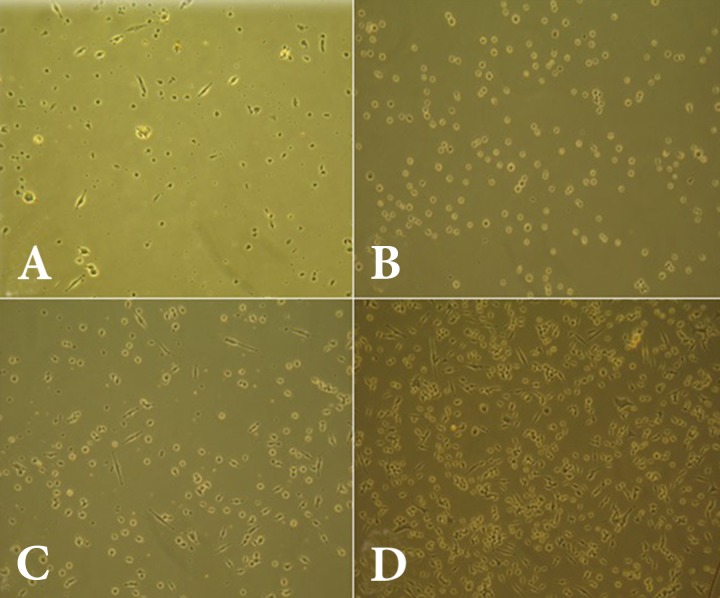
Cell viability among various dilutions of the Duraflur varnish; *A)* 1/2 dilution; *B)* 1/4 dilution; *C)* 1/8dilution; *D)* control

**Figure 4:**
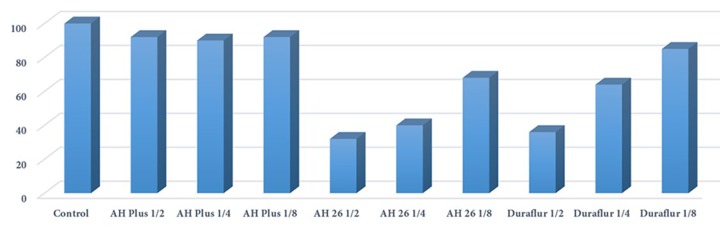
Cell viability in control and the test groups

All dilutions (1/2, 1/4, and 1/8) of the materials used in this study (AH-Plus, AH-26, and Duraflur) showed significantly lower cell viability compared to the control group (Figures 1 to 4). There was significantly higher cell viability in AH-Plus samples compared to AH-26 at all dilutions, while the viability of cells in AH-Plus samples was significantly higher compared to Duraflur at 1/2 and 1/4 dilution (*P*<0.001). Duraflur showed significantly higher cell viability compared to AH-26 at all concentrations except for 1/2 dilutions (*P*<0.001) (Table 1 and Figures 1 to 4).

**Table1:** Cell viability (%) in various concentrations of the tested materials

	**½ Concentration **			**¼ Concentration**			**⅛ Concentration **		
	**AH-Plus **	**AH-26 **	**Duraflur **	**AH-Plus **	**AH-26 **	**Duraflur **	**AH-Plus **	**AH-26 **	**Duraflur **
**Cell viability **	90.33	30.30	34.82	88.64	38.96	61.61	90.46	62.36	81.12

## Discussion

In this study, cytotoxic evaluation of the materials showed that despite higher cytotoxicity of Duraflur in comparison with AH-Plus except at 1/8 dilution, the material was significantly less cytotoxic than AH-26 at 1/4 and 1/8 dilutions (*P*<0.001).

In the present study, two root canal sealers, *i.e.* AH-Plus and AH-26, were compared with Duraflur varnish because of their extensive clinical application and also frequent employment in endodontic research as a golden standard to compare any newly introduced root canal sealer [9-18]. Cytotoxic evaluation of the test materials showed that despite higher cytotoxicity of Duraflur compared to the AH-Plus, it was significantly less cytotoxic than AH-26 at 1/4 and 1/8 dilutions. 

Resin-based sealers have some toxic effects which decrease over time as the concentration of leachable components is reduced [4]. AH-plus is a well-tolerated epoxy resin sealer in animal studies [5, 19]. Several investigations reported that AH-26 has higher cytotoxicity compared to AH-Plus [9-11], whereas others reported no significant difference between them in this regard [12-14]. The results of the present study showed lower cytotoxicity of AH-Plus.

Many cell lines have been used for evaluating the cytotoxicity of endodontic materials including mouse gingival fibroblasts, human osteosarcoma cell line [20], V79 ﬁbroblasts, murine granulocyte-macrophage progenitor cells [21], HGF [22, 23], Hela cells [24, 25] and fibroblasts of periodontal ligament [26]. In the present study, similar to several previous investigations, HGFs were used. 

Also several methods have been introduced for cytotoxicity testing of endodontic materials including: the 2, 5-diphenyl-SH-tetrazelium bromide colorimetric assay, *aka*. MTT assay, ﬂuorescent dyes and ﬂow cytometry. In the present study, MTT assay was used as a common technique for evaluating the cytotoxicity of dental materials [27-29]. MTT is a colorimetric assay for assessing cell viability. A yellow tetrazole [3-(4,5-dimethylthiazol-2-yl)-2,5-diphenyl tetrazolium bromide] is absorbed by the mitochondria where it is reduced to purple formosan by succinate dehydrogenase in living cells. An acidified solution is added to dissolve the insoluble purple formosan into a colored solution. The absorbance (OD) of this colored solution can be quantified by its measurement at a certain wavelength. By increased reduction of formazan and measurement of OD, cell viability and the cytotoxicity of materials can be measured [30]. In the present study three different dilutions (1/2, 1/4 and 1/8) of the tested materials were used as suggested by previous *in vitro* cell culture studies [23, 29].

A novel material for clinical use should always be evaluated by biocompatibility tests before introducing to the market [6]. Biocompatibility is evaluated at first through cell culture studies and at higher levels by intraosseous and subcutaneous implantation on animals [6]. Therefore, from the ethical point of view, it is wise to evaluate the cell toxicity prior to implantation investigations because in case the material shows higher cell toxicity compared to the currently used root filling materials, there would be no reason to evaluate them by implantation studies. In the present study, the culture of HGF was used and further implantation tests are required for evaluating biocompatibility and physical properties.

## Conclusion

In conclusion, as Duraflur has higher cell viability compared to AH-26, it can be assumed that the former material has potential as a root canal sealer. 
